# Voluntary assisted dying: estimating life expectancy to determine eligibility

**DOI:** 10.5694/mja2.51648

**Published:** 2022-07-24

**Authors:** Sharon H Nahm, Martin R Stockler, Belinda E Kiely

**Affiliations:** ^1^ NHMRC Clinical Trials Centre University of Sydney Sydney NSW

**Keywords:** Life expectancy, Survival analysis, Mortality, Suicide, assisted, Legislation, medical

Understanding what it means to identify people with less than 6 months to live

Victoria operationalised Australia’s first voluntary assisted dying (VAD) Act in June 2019, shortly followed by Western Australia. Since then, Tasmania, South Australia, Queensland and more recently New South Wales have passed similar Acts, with VAD to commence in all states in the next 18 months. The VAD Acts were designed to provide a safe legal framework for people with a terminal illness who wish to choose the manner and timing of their death. For a person to be eligible to access VAD, they must be diagnosed with a medical condition that is causing suffering that cannot be relieved in a manner that the person considers tolerable. In addition, eligibility requires doctors to document that the applicant’s life expectancy meets a particular criterion. There are differences among states in the exact wording of this requirement.

In Victoria and South Australia, a person may be eligible for VAD if they have a condition that is “expected to cause death within weeks or months, not exceeding 6 months”^1^, ^2^ and in Tasmania if the condition is “expected to cause death within 6 months”.^3^ The legislation in Western Australia, and New South Wales, states that the condition “will, on the balance of probabilities, cause death within a period of 6 months”.^4^, ^5^ In each of these states, a longer period (12 months) is allowed for people with a neurodegenerative disease. In Queensland, the legislation states that the condition is “expected to cause death within 12 months”,^6^ without distinguishing the type of condition. The wording of these eligibility criteria warrants careful examination and consideration.

For example, “on the balance of probabilities” is a legal concept, typically applied to the burden of proof in civil claims, generally taken to mean “more probable than not”, and therefore perhaps corresponding to a percentage probability of 51% or more. Does this mean that a person may be eligible for VAD if their probability of dying within 6 months is judged to be 51% or higher? The phrase “expected to die within 6 months” implies a higher degree of belief, but does this correspond to a probability of 70%, 80%, 90%, or some other percentage? The phrase “not exceeding 6 months” seems to imply an expectation that all people meeting this criterion would die within 6 months, corresponding to a probability of 100%. Despite these differences, we believe the intent behind the current legislation is that most people judged to be eligible for VAD would be expected to die within 6 months (or 12 months in Queensland).

Estimates of life expectancy are inherently uncertain and imprecise. To illustrate this, we pooled data from six studies of participants with a range of advanced cancers, in which we could compare their medical oncologist’s estimate of expected survival time (median survival time in a group of similar patients) versus the actual survival time that was subsequently observed.^7^, ^8^, ^9^, ^10^, ^11^, ^12^ We have previously reported that these estimates of expected survival time were imprecise, with less than 30% of participants having estimates within 0.67–1.33 times their observed survival time, but well calibrated (unbiased), with about equal proportions of participants living longer or shorter than their expected survival time.^7^, ^8^, ^13^, ^14^, ^15^ Of the 1057 participants, 182 had an expected survival time of less than 6 months, and of these, 127 (70%) died within 6 months. In other words, most patients in these studies with an expected survival time of less than 6 months died within 6 months.

These data have several limitations when applied to the issue of eligibility for VAD. Medical oncologists in these trials were asked to record “the expected survival time in a group of similar people”, not whether they “expected death to occur within 6 months”. Furthermore, participants in cancer clinical trials would be expected to have longer survival times than people seeking assistance with dying. Finally, our data are confined to people with advanced cancer, and we have no data about prognostication in people with other terminal illnesses. Despite these limitations, our findings support the claim that Australian medical oncologists participating in these studies were reasonably good at predicting a survival time with a probability of 50%, even if they were unable to accurately predict each individual’s survival time.

The VAD legislation requires doctors to predict an unspecified probability of a patient dying within a certain period. This is different to the question more commonly asked by patients, which is “how long have I got?”. Doctors are not trained to formulate estimates of expected survival time, or to explain them to patients. We predict that many doctors will find it difficult to answer whether they expect individual patients to die within 6 months.

Our research on prognostication in advanced cancer has shown that ranges of survival time corresponding to information sought by patients, namely a worst‐case scenario, a typical scenario, and a best‐case scenario, can be determined using simple multiples of an oncologist’s estimate of the expected survival time (ie, median survival time in a group of similar patients). For example, when medical oncologists were asked to estimate the expected survival times for an individual patient in our studies, about 5–10% of individuals died within one‐quarter of their expected survival time (worst‐case scenario), the middle 50% lived from half to double their expected survival time (typical scenario), and 5–10% lived longer than three times their expected survival time (best‐case scenario).^7^, ^8^, ^13^, ^14^, ^15^, ^16^

The wording of laws about VAD does not clarify how certain a doctor should be that an individual requesting VAD would die within the specified time. If we take “expected to die within 6 months” to mean an expectation that about 90% of such people would die within 6 months, then this corresponds to a best‐case scenario of 6 months. People eligible for VAD would therefore be those with an expected survival time of 2 months (one‐third of 6 months) or less. Among a group of individuals with expected survival times of 2 months, we would expect 5–10% to die within 2 weeks (one‐quarter of 2 months), 50% to live 1–4 months (half to double 2 months), and 5–10% to live beyond 6 months (three times 2 months). Similarly, individuals with an expected survival time “not exceeding 12 months” (as per the Queensland legislation) would be those with an expected survival time of 4 months or less, among a group of whom we would expect 5–10% to die within 1 month, 50% to live for 2–8 months, and 5–10% to live beyond 12 months. We wonder whether the intention behind the current legislative wording is that people eligible for VAD are those who are unlikely to survive beyond 6 months, meaning a best‐case scenario of 6 months and an expected survival time of 2 months.

We are not advocating that the eligibility criteria be broadened or narrowed, rather, our aim is to highlight our uncertainty about which patients are eligible for VAD under current legislation. Assessing a person’s eligibility for VAD is difficult because prognostication is difficult, prognosis is inherently uncertain, and the eligibility criteria are not clearly specified. Legislation should be improved by including clearer definitions and explanations of phrases such as “expected to cause death within 6 months” using probabilistic terminology that corresponds with how prognoses are best formulated and communicated.

## Open access

Open access publishing facilitated by The University of Sydney, as part of the Wiley ‐ The University of Sydney agreement via the Council of Australian University Librarians.

## Competing interests

No relevant disclosures

## Provenance

Not commissioned; externally peer reviewed.
